# Before the 2020 Pandemic: an observational study exploring public knowledge, attitudes, plans, and preferences towards death and end of life care in Wales

**DOI:** 10.1186/s12904-021-00806-2

**Published:** 2021-07-20

**Authors:** Ishrat Islam, Annmarie Nelson, Mirella Longo, Anthony Byrne

**Affiliations:** 1 Marie Curie Palliative Care Research Centre, School of Medicine, Cardiff University, Cardiff, CF14 4YS UK; 2 Department of Palliative Medicine, Velindre NHS Trust, Cardiff, CF15 7QZ UK

**Keywords:** End of Life Care, Death and dying, Public attitudes, Public health, Supportive care, Terminal care, Cultural issues, Communication, Quality of life

## Abstract

**Background:**

Understanding public attitudes towards death and dying is important to inform public policies around End of Life Care (EoLC). We studied the public attitudes towards death and dying in Wales.

**Methods:**

An online survey was conducted in 2018. Social media and the HealthWiseWales platform were used to recruit participants. Data were analysed using descriptive statistics and thematic analysis.

**Results:**

2,210 people participated. Loss of independence (84%), manner of death, and leaving their beloved behind were the biggest fears around death and dying. In terms of EoLC, participants sought timely access to care (84%) and being surrounded by loved ones (62%). Being at home was less of a priority (24%). Only 50% were familiar with Advance Care Planning (ACP). A lack of standard procedures as well as of support for the execution of plans and the ability to revisit those plans hindered uptake. The taboo around death conversations, the lack of opportunities and skills to initiate discussion, and personal fear and discomfort inhibited talking about death and dying. 72% felt that we do not talk enough about death and dying and advocated normalising talking by demystifying death with a positive approach.

Health professionals could initiate and support this conversation, but this depended on communication skills and manageable workload pressure. Participants encouraged a public health approach and endorsed the use of: a) social media and other public platforms, b) formal education, c) formal and legal actions, and d) signposting and access to information.

**Conclusions:**

People are ready to talk about death and dying and COVID-19 has increased awareness. A combination of top-down and bottom-up initiatives across levels and settings can increase awareness, knowledge, and service-utilisation-drivers to support health professionals and people towards shared decisions which align with people’s end of life wishes and preferences.

**Supplementary Information:**

The online version contains supplementary material available at 10.1186/s12904-021-00806-2.

## Background

Contemporary Western societal approaches to death have been criticised for marginalising death and characterising it as a “forbidden subject” [1]. This has hindered open conversations about death and End of Life Care (EoLC), despite UK research and policy [2–8] mandating the importance of effective conversations about the end of life [8–10]. Cox and colleagues’[11] systematic review focusing on public attitudes towards death and dying in the UK has highlighted key concerns of fear of the unknown, experiencing distress, and becoming a burden to the family. ‘Understanding Dying’ was also identified as a theme in the James Lind Alliance national survey process of identifying the top ten research priorities for palliative and End of Life care [12]. A supplementary analysis of participants’ narratives [13] emphasised a general lack of understanding of death and dying processes and the frustrations of participants when faced with a situation for which they felt unprepared. The need to reduce the uncertainty of prognostication, a better understanding of what to expect, and how to recognise when death is near to allow time for appropriate preparations were key concerns as well as the importance of supporting the wellbeing of staff, patients, and their families.

Death and EoLC is therefore clearly a public health issue [14] with its high burden, social impact, and the need to prevent suffering. In the UK and worldwide [15–17], there have been campaigns and initiatives to raise public awareness and facilitate discussions around death.

The COVID-19 pandemic has suddenly brought the reality of death to the forefront of public consciousness and highlighted new circumstances around death including the forced separation of patients and families at end of life, the challenges of future care decision making, and conversations on treatment escalation and place of care when dying [18–21]. The pandemic has highlighted death and dying as topics of immediate concern in our daily lives, forcing the way we think about death to re-assimilate at pace in what is likely to provoke a longstanding societal shift.

Public preference is vital in shaping effective palliative and EoLC services [2, 22]. Pre COVID-19 public data can be useful to determine the baseline in terms of how the public feels about death and dying and determine what, under the current circumstances, might be prioritised for public engagement. This paper describes the findings from a public survey carried out in 2018 with the aim of understanding people’s attitudes towards death and dying in Wales. The specific objectives were to learn about people’s:

- Fears about death and dying
- Preferences and priorities around EoLC
- Knowledge around the terminologies commonly used in EoLC and understanding about Advance Care Planning (ACP)
- EoLC plans
- Communication around death and dying

Most previous research has used either numerical or narrative data. In this study, we aimed to build on previous findings and use comments from open-ended questions to integrate, interpret, and validate the findings emerging from the closed questions.

## Methods

### Study design and setting

**Fig. 1:**
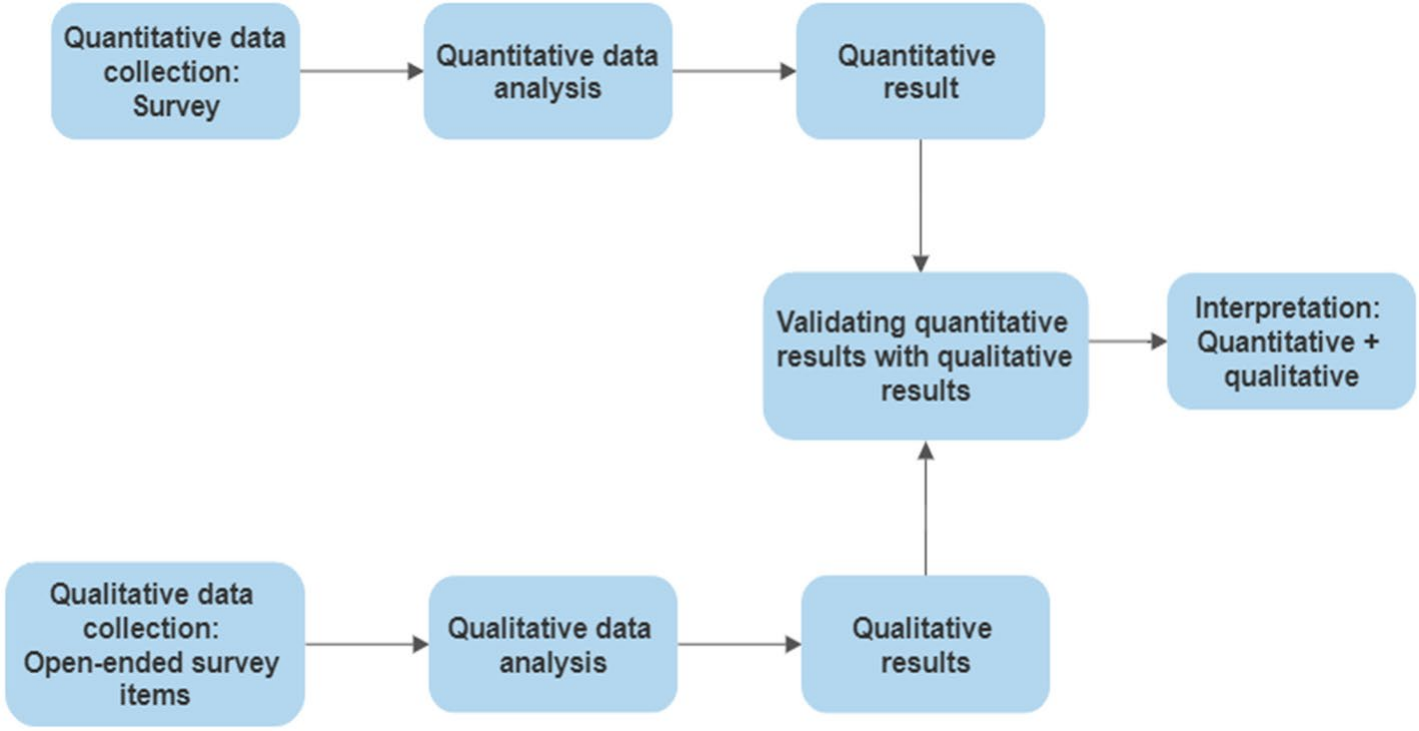
The Triangulation Design for Validating quantitative data model

This study is a cross-sectional observational study using the Triangulation Design: Validating quantitative data model of mixed-method approach in data analysis (Fig. 1). In this model, both quantitative and qualitative data are collected within one survey instrument. This is an established model to use when open-ended questions are created as add-ons to a quantitative survey and quantitative findings are validated and expanded on with the qualitative findings [23].

### Patient and public involvement

Patient and Public Involvement (PPI) adhered to the framework for the six UK standards of public involvement were followed (‘working together’, ‘communication’ and ‘impact’) [24]. Seven lay research partners from the known networks were engaged throughout the development and piloting of the survey questionnaire. Regular contacts were maintained via emails, telephone calls, and face-to-face conversations to incorporate their inputs into the different versions of the questionnaire. In addition to this, one of the research partners contributed to the rest of the study by identifying potential recipients for the questionnaire and commenting on the dissemination material.

The survey was conducted in Wales between 31/01/2018 to 21/05/2018. Self-selected participants responded using the Jisc Online Survey tool.

### Participants

Adults residents in Wales and with the capacity to consent were eligible for the study. An invitation to take part in the study and the link to the online questionnaire were advertised via social media and institutional websites. In addition, email invitations were sent to public and private organisations (Supplementary File 1: STROBE [25] checklist). In 2015 the National Council for Palliative Care commissioned ComRes, a member of the British Polling Council, to conduct a similar survey where 2,016 adults from the UK were interviewed online. A similar quota of participants was anticipated for this study, 123 respondents [2].

### Recruitment strategy

A stakeholder mapping exercise was conducted to reach adult residents in Wales and with the capacity to consent. Posts with the survey link were created using mainly Twitter and Facebook. Requests to retweet or share the post were sent via tweet to influential colleagues, all relevant public organisation across Wales (NHS Trusts, University Health Boards, Public Health Wales, Government bodies, local councils, universities, charities, hospices, care homes, and superstores). Emails were sent to the press, media, and communication team of relevant public organisations. All recipients were encouraged to disseminate the link among friends and families.

Healthwise Wales is a unique platform that has 28,000 registered members interested in taking part in health research across Wales and a link to the survey was sent to its members.

### Questionnaire development

A literature scoping process identified previous studies to inform the conceptual design. A questionnaire was then developed and tested for content and face validity by members of the research team. Relevant questions were adapted from existing survey questionnaires where possible including the Dying Matter Coalition [2] and the VOICE survey [26]. In addition, the findings from the PeolcPSP study [12] helped inform some of the questions and the questionnaire. A panel of 10 topic experts and three colleagues with no expertise on this subject reviewed the draft of the questionnaire. The revised questionnaire was further validated by eight academics and seven research partners.

The final draft of the questionnaire covered six sections and included open and closed questions (with Likert scales) aiming to gauge participants’ i) views about death, ii) feelings about death and dying, iii) knowledge on commonly used terms in EoLC and relevant facilities, iv) preferences in EoLC, v) plans around death and dying, and vi) socio-demographic information. A consent statement followed a summary of the eligibility criteria, anonymisation issues, and data management (Supplementary File 2: Survey Questionnaire).

### Consent

Participants were self-selected and proceeded to the survey if they agreed to the consent statement. After consenting, participants could avoid responding to any of the questions if they wished). A Welsh version of the questionnaire was available on request.

### Data analysis

Descriptive statistics were used to analyse the numerical data. We studied the representativeness of the study sample by comparing the characteristics of the study sample to the Welsh population statistics. The pattern of missing data was investigated. IBM SPSS statistical software package (V. 25) was used to run the analysis.

The comments from the open-ended questions were analysed using Thematic Analysis [27]. Following the six steps, the researchers first familiarised themselves with the data. The comments were then systematically coded following a pattern of similar ideas whilst ensuring data relevant to these codes were collated across all survey data. Themes were derived in a deductive manner using the questions as a coding framework. Subsequent themes and subthemes were derived along the analyses using an inductive approach. A second independent coder validated the coding. Themes and subthemes (including any discrepancy in the coding) were further refined and reviewed following discussions with the study team to avoid any observers’ bias. Verbatim quotes were used throughout the report as illustrative examples. NVivo (V. 12) was used to manage and catalogue the data. Given the mixed methods approach, mixed-methods [28] and survey [29] study reporting guidelines are used throughout the paper.

## Results

From 31/01/2018 to 21/05/2018, a total of 2,210 people responded to the survey. 12 requested the Welsh version. The sample was representative of all geographical areas across Wales. Males, people with chronic illness and disability, and people with a lower level of education were underrepresented (Supplementary File 3: Table S1). The participants’ circumstances at the time of the study are listed in Table 1.

**Table 1:** Circumstances of study participants (n = 2204)

	Number	Percent
I am a bereaved carer/ family member/ partner/ spouse/ friend who lost a loved one in the last 5 years	342	15.5
I am a carer/ family member/ partner/ spouse/ friend of someone who is thought to be in the last few years of their life	190	8.6
I am a health / social care professional (non-clinical)	122	5.5
I am a health care professional (clinical)	204	9.3
I am a health researcher/ an academic who has an interest in the subject	59	2.7
I am a member of the public who has an interest in the subject	949	43.1
I am a professional working with people who are thought to be in the last few years of life	99	4.5
I am a volunteer working with people who are thought to be in the last few years of life	16	0.7
I consider myself to be in the last few years of my life	107	4.9
Other	116	5.3

Overall, missing data was negligible (below 2%). Around 10% of the participants did not answer two questions: having a chronic mental illness and if moved from another country.

**Fig. 2:**
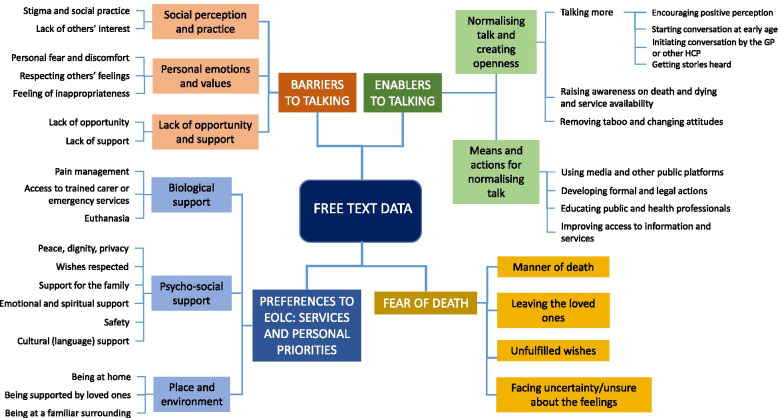
Themes and subthemes from the analysis of the free text data

The participants’ comments merged under four main themes: enablers and barriers to talking about death and dying, fear of death, and EoLC preferences in terms of services and personal priorities (Fig. 2). A sample of quotations from each theme is reported in Supplementary File 3: Table S2.

To ease the presentation of the results, the answers to the closed questions are integrated, contextualized, and validated with the themes emerging from the participants’ comments.

### Fears about death and dying

The fear of being helpless and dependent was overwhelming across participants’ responses (Supplementary File 3: Fig S1). In their comments, participants expressed their reasons for fearing death, these included: the manner of death, experiencing suffering and pain, losing dignity, facing the unknown, leaving wishes and deeds unfulfilled, and leaving loved ones.

*“I don’t fear death but the manner of dying” (PID 32)*

*“If it happened now—where would my children live?” (PID 404)*

### Preferences and priorities around death and EoLC

**Fig. 3:**
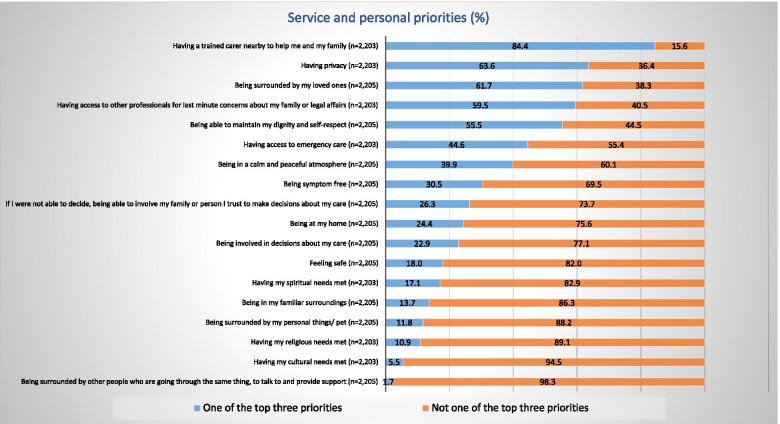
Participants’ preferences for EoLC services and personal priorities

The majority of the participants would wish to gain quality of life over survival (Supplementary File 3: Fig S2). In terms of EoLC services and personal priorities, participants showed a strong focus on being able to access care provision for themselves and their families and being surrounded by their loved ones (Fig. 3).

Similar to the numerical data, the participants’ comments strongly converged towards aspects of service provision (i.e. timely access, peaceful environment) and presence (and wellbeing) of family and friends rather than a physical place of care.

*“Having someone to discuss how end of life meds and treatment is administered” (PID 576)*

*“I'd like to be kept alive until my family can get to me to say goodbye! One lot lives in Scotland, the other in Australia” (PID 1262)*

*“The calm space to say goodbye well, and support to ensure everything that needs to be asked has been” (PID 1001)*

*“Knowing that there is someone there to give support to my loved ones during my illness/following my death” (PID 758)*

Some wanted to avoid the burden and decisional conflict for families, particularly where capacity is lost.

*“My family's wishes would take priority over mine - I don't want them to be more upset than necessary. This is particularly so if I have advanced dementia and am not aware of what is happening” (PID 214)*

### Knowledge around EoLC and understanding of Advance Care Planning (ACP)

**Fig. 4:**
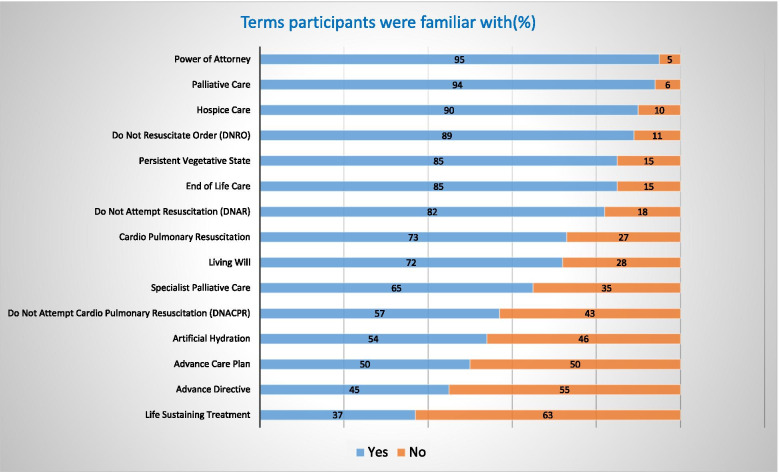
Participants’ familiarity with EoLC terminology

The majority of the respondents were very familiar with many terms related to EoLC, however, only 50% and 45% were familiar with ACP and Advance Directives, respectively (Fig. 4).

Participants expressed doubts about their wishes being respected and frustration about the absence of a formal procedure or standard practice to deal with advanced care plans in the healthcare settings.

*“Then it is important that they are listened to and not ignored. My mother's medical power of attorney was ignored as the care home staff didn’t agree with it”* (PID 366)

*“Yes – BUT [there] needs to be a method of communicating this to the professionals for complete security. Too many Living Wills are ignored or are unknown”* (PID 287)

Participants were confused about the appropriate timing for preparing the plan and opportunity of reassessment without which the plan might lose its relevance and efficacy.

*“I think it's ok to state your preferences in advance but there must be flexibility in the system because circumstances change, and these changes could affect the decisions one has made.”* (PID 400)

Overall participants acknowledged that ACP would help increase their autonomy of choice whilst avoiding any unwanted treatment.

*“Having a sense of control over how I will be treated and peace of mind that my wishes will be considered and respected.”* (PID 56)

**Fig. 5:**
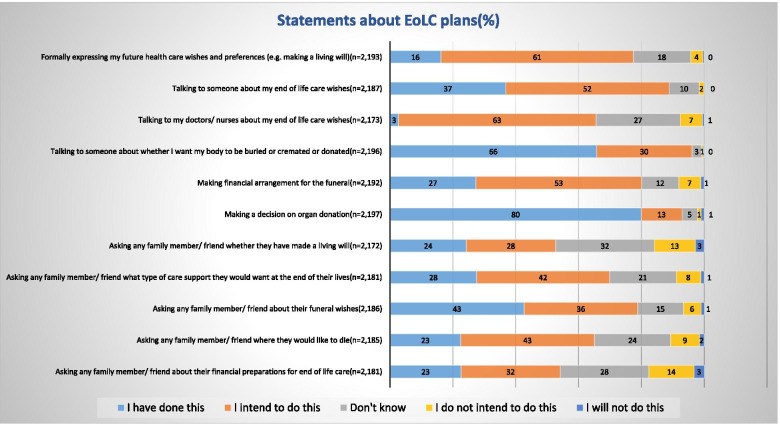
Participants’ statements about EoLC plans

In practice, only 16% of the sample had formalized any EoLC plans such as Living Will (Fig. 5), by contrast, 61% indicated their intention to do so.

Notably, 80% of the participants had formalized their decision around organ donation, which might be related to the opt-out system introduced in Wales in 2015.

### Communication around death and dying

The majority of participants felt ready to talk about death and dying and valued strongly a sense of control and choice over EoL treatment and care, personal arrangements (e.g. financial) (Supplementary File 3: Fig S3) and life-sustaining technologies (Supplementary File 3: Fig S4).

**Fig. 6:**
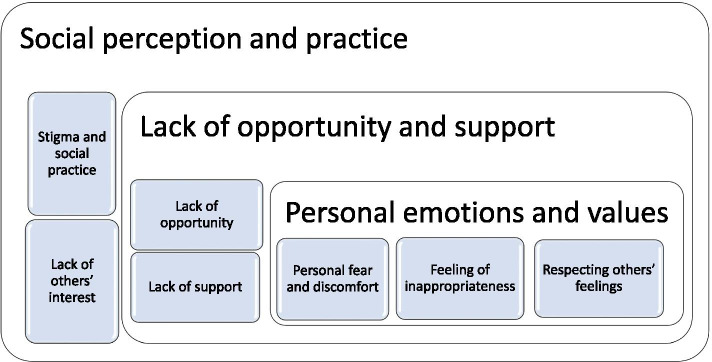
Factors that inhibit talking about death and dying

However, 72% thought that, as a community, we do not talk enough about death and dying. Three main factors (Fig. 6), acting at three different levels, prevent conversations about death and dying: i) social perception and practice, ii) lack of opportunities and support, and iii) personal emotions and values.

#### Social perception and practice

Participants believed that people would not be interested in talking about death as this was not socially appropriate. Talking about death was considered a social taboo and perceived as impolite or morbid.

*“Upsetting subject for others. Uncomfortable to think about—our society teaches us that death is a taboo subject” (PID 872)*

#### Lack of opportunities and support

Participants found it hard to find the right time to talk about death or did not feel they had family members and friends with whom they would talk.

*“Too busy living right now, not a priority” (PID 599)*

Others expected support from health professionals but felt that time pressure or lack of communication skills did not allow for sensitive discussions. It was also feared that discussions would be documented. Lack of information around available services hampered these discussions as well.

*“From previous experience Healthcare professionals often find it difficult to discuss” (PID 309)*

#### Personal emotions and values

Many participants acknowledged their fear and discomfort around talking about death and dying, especially when still grieving for the death of someone close. The fear of hurting other people’s feelings, especially those of family members, prevented initiating the discussion.

*“I'm scared of dying and talking about death makes me anxious” (PID 779)*

**Fig. 7:**
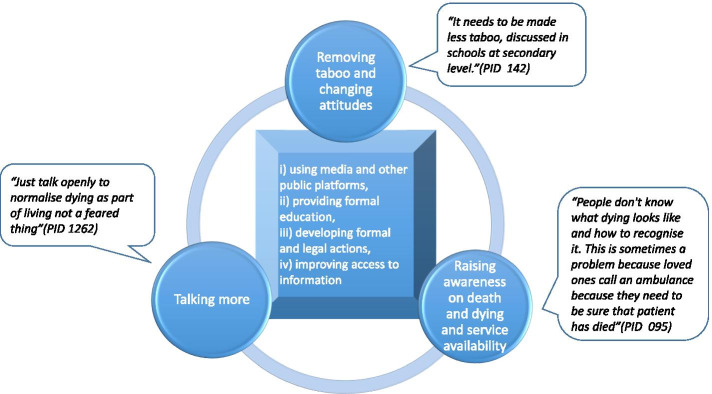
Normalising talk about death and dying – focus and mode

Participants identified opportunities to improve talking about death with normalisation emerging as an overarching theme, subthemes are summarised in Fig. 7.

Participants believed that a cultural shift around the negative perception of death with a focus on demystifying death and a more positive approach to encouraging societal conversations are needed, and this shift would enhance the acceptance of death as a part of life. In an attempt to break the stigma around death, participants advocated a public health approach by including death and dying as part of school curricula, using national print, TV and social media, and other public platforms to promote conversations and more open signposting to existing EoLC and ACP services. GPs and other health and social care professionals could prompt discussions to avoid very late conversations with unprepared patients and families. This would be made easier by raising awareness within communities around issues such as palliative and End of Life Care, funeral arrangements, bereavement services, organ donations, and living will and help them to acquire the necessary competency to empower their decision making around planning for care at the end of life. Notably, some suggested adopting more coercive approaches such as making funeral plans compulsory and ACP universal in care-homes settings. A limited knowledge around where to retrieve the relevant information was also evident (Supplementary File 3: Fig S5).

## Discussion

We used an online survey to study people’s knowledge, attitudes, plans, and preferences towards death and EoLC in Wales. A total of 2,210 participants took part in the study. The participants contributed 2,610 comments across eight open-ended questions.

Participants especially feared specific aspects linked to the process of dying such as the loss of independence (84%), the manner of death (comments), and having to leave their beloved behind (54%).

In terms of service provision and personal EoLC preferences, participants favoured aspects of service provisions such as timely access to care (84%) and being surrounded by family and friends (62%). Being at home was less of a priority (24%).

Only 50% of the sample were aware of ACP. ACP was perceived as a means to increase the autonomy of choice around EoLC and to avoid unwanted treatment (comments). However, this depended on the presence of standard procedures, support for the execution of plans, and the ability to revisit plans (comments). These factors might explain why a large proportion of respondents intended to arrange EoLC plans but, except for organ donation, only a minority had formalized any EoLC plans.

Communication around death and dying were hindered by the taboo around death conversations, the lack of opportunities and skills to initiate discussion, and personal fear and discomfort. Respondents advocated normalizing talking about death and dying—a cultural shift encouraged by demystifying death with a positive approach. Health professionals can initiate and support this conversation, but communication skills and manageable workload pressure were crucial for facilitating this.

Participants sought a public health approach to breaking the stigma around death and dying and endorsed the use of four channels to achieve this: a) using social media and other public platforms, b) providing formal education, c) developing formal and legal actions, and d) improving signposting and access to information.

The study findings provide robust estimates to corroborate previous research in terms of people’s fears around the process of dying [11–13] and reinforces the evidence [30] that the preferred place of death is associated with specific environmental factors. Again, similarly to the Dying Matter Coalition survey [2], nearly 90% of the respondents felt comfortable discussing issues around death, but the percentage of people who had started any discussions with health care professionals remains small (a change from 2 to 3%). However, there was an increase from 7.5% to 16% in the number of people formally expressing future health care wishes. The thematic analysis indicates that people would welcome the GP or other health care professional (HCP) to initiate the conversation. However, the same data suggests that people may not feel completely supported by HCPs and that workload pressure and lack of communications skills were highlighted as some important deterrent factors. The journey of a person in EoLC can be characterized by several moves across different healthcare providers/agencies, and patients’ trust and engagement towards the different HCPs might vary. A full understating of the micro and macro factors that support patients, families, and HCPs initiating discussion around EoLC planning was beyond the scope of this study. There is also growing evidence around the role that non-healthcare platforms can play in initiating discussions around EoLC. In 2020 McDonnell and Idler completed a literature review on promoting ACP among African American faith communities and advocated that involving faith leadership, exhibiting cultural competency, preserving a spiritual/Biblical context, were some of the key factors for a successful ACP program implementation [31].

More recently, a mixed-method study [32] explored attitudes to discussing death and dying amongst adults with an advanced or terminal condition. For this study population, the authors conclude that death was far from taboo; instead, they found talking about death and dying “liberating”. Participants sought honest and open conversations about their choices and, similarly to our study, they expressed a need for more support to achieve what they define as a good death; including the choice of a safeguarded assisted dying law alongside good palliative care. This could in part explain why only 12% of the respondents had completed an Advance Decision to Refuse Treatment. The uptake of ACP remains low outside the UK as well. Yadav and colleagues [33] conducted a systematic review of the prevalence of Advance Directives (AD) among US adults and found that only one in three adults had carried out some form of AD. A similar percentage applied to residents of long-term care facilities across European countries [34].

Barriers to a higher uptake of ACP include scepticism about wishes being respected [35], social grade [36], communication skills and competency [35, 37–39]. Abel and colleagues (2020) sustain that a shift of focus from medical treatments to health and wellbeing would facilitate an early engagement and ease this difficult conversation across all care settings [40].

Participants seek a public health approach to normalise talking about death [3–5]. An effective public health programme should include an educational component, a community component, and a government/service role [41]. Initiatives such as Dying Matters in Wales or installation of Departure Lounge in England [42] are actively working towards this. In Wales, a Clinical Lead and ACP facilitators at the Health Boards and charities such as Macmillan , Marie Curie, and Byw Nawr work towards raising public awareness of ACP in the community and educating facilitators and health care professionals. In 2015, the UK statistics indicated that a child loses a parent on average every 22 min [43]. Bereavement can lead to social isolation and further consequences such as alcoholism, depression, and antisocial behaviour. This loss of human capital is, at least in part, due to unacknowledged loss. In some countries, death education is part of the school curricula [44]. In the UK, this is currently limited to programs of support for bereaved young people [43].

As is the case for other online survey-based studies, this study has strengths and limitations. The large sample allowed for robust estimates and rich comments. An extensive literature review, questionnaire piloting, and PPI support secured the questionnaire content and face validity. From the analysis of comments to open-ended questions, it emerged their lived experience in a free-ranging discourse originated from their interactions with close ones or health care providers. The qualitative data provided additional dimensions and validations of the numerical findings. The survey was online and, in 2018, around 10% of the adult population of Wales reported not using the internet. Male, singles, and people with a lower educational background were under-represented and future research should consider oversampling from these groups to address any sample selection bias. Also, participation might be affected by a selection bias because only people who were interested in the topic might be more likely to take part. The high percentages of participants agreeing to the attitudinal answers together with the large sample generated robust estimates; however, these might not apply to people underrepresented in the sample such as ethnic minority groups.

### The impact of COVID-19

COVID-19 has severely altered the health environment and transformed the delivery of care. Health professionals might be unable to rely on customary norms to create rapport and continuity of care: Consultations occur through screens and protective equipment, communication with families and friends is done remotely, and difficult conversations might have to take place in emergency contexts, shortly after meeting the patient, because disease progression is such that the patient needs to be diverted to the EoLC pathway. However, today’s technology can support this consultation mode [45]. In Wales, the NHS Wales Video Consulting (VC) Service is a video consultation service [46] rolled out by Welsh Government to offer video consultations where possible, the system has currently covered 90% of GP practices in Wales.

The daily updates on mortality counts and rates are crucial in highlighting the public health message and have instilled a deep awareness around death and dying. However, six months into the COVID-19 pandemic, mass-media headlines continue to focus on these statistics only. There is now a need to re-direct the conversation and pay attention to the process of dying and how we make sense of mortality.

In the UK and across the world, the pandemic has inevitably propelled Palliative Care (PC) centre stage [21] and turned it into a driver of best practice[47] to ensure access to EoLC is in respect of patients’ priorities and preferences. The PC philosophy is becoming the vehicle to safeguarding patients’ autonomy and avoiding decisions being driven by the fear of the pandemic [48]. However, this is only possible if PC becomes more visible, starting with national (and international) guidelines and policy documents clearly embedding the contribution of PC when treatments no longer aim to cure and shift to comfort.

In the UK a group of independent charities, health and social care bodies and research organisations initiated the 'What matters' movement aiming to promote a culture that enables personal wishes to be heard. In Wales, alongside the NHS, organisations from the third sector such as Marie Curie, Macmillan, Dying Matters, Byw Nawr, Hospice Care, Cruse, and Compassionate Communities are playing an important role in educating the general public and HCPs on how to initiate talking about death, prepare an ACP, and express EoLC preferences as well as improving understanding of the bio-psychosocial, cultural and spiritual aspects of needs and wishes at the end of life. They advocate that the endorsement of this cultural change should be everybody’s responsibility including the public, HCPs, carers, faith leaders, educators, and policy-makers. The development of appropriate conversational tools applicable across healthcare settings might encourage individualistic stances [49].

COVID-19 has also created an unprecedented number of fast-tracked research, which highlights the need to test and share examples of best practice as soon as they become available. A follow-up survey is underway to remedy some of the sample bias [49, 50] and to gauge real-time data about how COVID-19 has affected people’s attitudes towards, and engagement with, EoLC planning.

## Conclusions

The findings resonate with and reinforce knowledge from before the pandemic: people are ready to talk about death and dying, and COVID-19 has acutely increased awareness of this topic. Participants advocate the adoption of a public health framework and acknowledge the need for a combination of top-down and bottom-up initiatives across levels and settings to increase awareness, knowledge, and service utilisation. This would encourage earlier discussions and preparation of advance care plans and support health professionals and people towards shared decisions which closely align with people’s end of life wishes and preferences.

## Supplementary Information

** Supplementary file 1**. STROBE checklist.

**Supplementary file 2**. Survey Questionnaire.

**Supplementary file 3**. Tables and Figures.

## Data Availability

The datasets used during the current study are available from the corresponding author on reasonable request.

## Abbreviations

ACP: Advance Care Planning
AD: Advance Directives
EoL: End of Life
EoLC: End of Life Care
PC: Palliative Care
PID: Participant’s ID
PPI: Patients and Public Involvement
SMREC: School of Medicine Research Ethics Committee
VC: Video Consultation
