# Supplementary material for: Before the 2020 Pandemic: an observational study exploring public knowledge, attitudes, plans, and preferences towards death and end of life care in Wales
Source: BMC Palliat Care. 2021 Jul 20;20:116. doi: 10.1186/s12904-021-00806-2 (PMC8290392; doi:10.1186/s12904-021-00806-2)
Supplement: Supplementary file 1 — STROBE checklist. [file 12904_2021_806_MOESM1_ESM.docx]

**SUPPLEMENTARY FILE 1:**

STROBE Statement

Knottnerus A, Tugwell P. STROBE--a checklist to Strengthen the Reporting of Observational Studies in Epidemiology. Journal of clinical epidemiology. 2008 Apr 1;61(4):323.

|  | Item No | Recommendation | Page  No |  |
| --- | --- | --- | --- | --- |
| **Title and abstract** | 1 | (*a*) Indicate the study’s design with a commonly used term in the title or the abstract | Cited on page #1.  Under the section ‘Title’. |  |
|  |  | (*b*) Provide in the abstract an informative and balanced summary of what was done and what was found | Cited on page #1.  Under the section ‘Abstract’.  The abstract provides a balanced summary of the study aim, method and findings. |  |
| Introduction | | | |  |
| Background/rationale | 2 | Explain the scientific background and rationale for the investigation being reported | Cited on page #2-3.  Under the section ‘Introduction’.  The study rationale has been established and contextualized by providing information on work done in the relevant field and by identifying research gaps. Also, the relevance of the study topic to the COVID-19 pandemic is explained. |  |
| Objectives | 3 | State specific objectives, including any prespecified hypotheses | Cited on page #3.  Under the section ‘Introduction’.  Study aims and objectives are listed. |  |
| Methods | | | |  |
| Study design | 4 | Present key elements of study design early in the paper | Cited on page #3.  Under the section ‘Study-design’. |  |
| Setting | 5 | Describe the setting, locations, and relevant dates, including periods of recruitment, exposure, follow-up, and data collection | Cited on page #3.  Under the section ‘Study-design’. |  |
| Participants | 6 | (*a*) *Cohort study*—Give the eligibility criteria, and the sources and methods of selection of participants. Describe methods of follow-up  *Case-control study*—Give the eligibility criteria, and the sources and methods of case ascertainment and control selection. Give the rationale for the choice of cases and controls  ***Cross-sectional study***—Give the eligibility criteria, and the sources and methods of selection of participants | Cited on page #3.  Under the section ‘Participants’.  An invitation to take part in the study and the link to the online questionnaire was shared via the Twitter accounts of influential colleagues and many public and private organizations. Two reminders to retweet the link were sent to fifty-one organizations, including University Health Boards, universities in Wales, and the ‘Dying matters’ Coalition. Cardiff University, HealthWiseWales, Marie Curie Research Centre, and the Welsh Cancer Research Centre shared the link via their organizational website. | |
|  |  | (*b*) *Cohort study*—For matched studies, give matching criteria and number of exposed and unexposed  *Case-control study*—For matched studies, give matching criteria and the number of controls per case |  | |
| Variables | 7 | Clearly define all outcomes, exposures, predictors, potential confounders, and effect modifiers. Give diagnostic criteria, if applicable | Cited on page #4.  Under the section ‘Questionnaire development’.  We used both closed and open-ended questions. Closed questions were mainly attitudinal questions presented in Likert-scale format. Most of the questions were based on previously used questionnaires. | |
| Data sources/ measurement | 8* | For each variable of interest, give sources of data and details of methods of assessment (measurement). Describe comparability of assessment methods if there is more than one group | Cited on page # 4.  Under the section ‘Data analysis’.  For open-ended questions, thematic analysis was used to analyze the qualitative data.^1^  Except for socio and demographic information, the quantitative data were collected using the Likert scale questions.  Mixed-methods study reporting and survey study reporting guidelines are used throughout the paper.^2, 3^ |  |
| Bias | 9 | Describe any efforts to address potential sources of bias | Cited in Table S1 of the Supplemental File 1.  We described the bias in terms of sample representativeness |  |
| Study size | 10 | Explain how the study size was arrived at | Cited on page # 4.  Under the section ‘Participants’.  The initial sample size was based on similar research. The study was online. We estimated that at least 123 respondents would need to be recruited, considering the total population in Wales and aiming to reflect the same proportion of respondents in a similar survey in England. A total of 2,210 participants took part in the survey. This allowed for more robust estimates. |  |
| Quantitative variables | 11 | Explain how quantitative variables were handled in the analyses. If applicable, describe which groupings were chosen and why | Cited on page # 4.  Under the section ‘Data Analysis’.  Quantitative data in the form of Likert scales were grouped to ease analysis, e.g. very comfortable and comfortable. Descriptive statistics were used to analyze the data.  No statistical comparisons were carried out.  We used comparisons to study the pattern of missing data. |  |
| Statistical methods | 12 | (*a*) Describe all statistical methods, including those used to control for confounding | Cited on page # 4.  Under the section ‘Data Analysis’.  Regression analysis was used to study the pattern of missing data |  |
|  |  | (*b*) Describe any methods used to examine subgroups and interactions | N/A |  |
|  |  | (*c*) Explain how missing data were addressed | Cited on page # 4  We quantified the amount of missing data for each question and checked the random distribution of missing data. Tables and figures report the data available for each variable and each dimension of the Likert scale questions. |  |
|  |  | (*d*) *Cohort study*—If applicable, explain how loss to follow-up was addressed  *Case-control study*—If applicable, explain how matching of cases and controls was addressed  *Cross-sectional study*—If applicable, describe analytical methods taking account of sampling strategy | Cited on page # 3  Under the section ‘Study design’.  This is a study aiming to explore public attitudes and as such, the sampling strategy was “random sampling” of the population, using an online questionnaire that was widely distributed and made accessible. |  |
|  |  | (*e*) Describe any sensitivity analyses | N/A |  |

Continued on next page

| Results | | | |
| --- | --- | --- | --- |
| Participants | 13* | (a) Report numbers of individuals at each stage of study—eg numbers potentially eligible, examined for eligibility, confirmed eligible, included in the study, completing follow-up, and analysed | 2,210 participants, the study did not include follow-up. |
|  |  | (b) Give reasons for non-participation at each stage | N/A |
|  |  | (c) Consider use of a flow diagram | N/A |
| Descriptive data | 14* | (a) Give characteristics of study participants (eg demographic, clinical, social) and information on exposures and potential confounders | These are listed in Supplement, File 1, Table S1. |
|  |  | (b) Indicate number of participants with missing data for each variable of interest | These are summarised on page #6, in addition to this, figures and tables include the data available for each data variable presented. |
|  |  | (c) *Cohort study*—Summarise follow-up time (eg, average and total amount) | N/A |
| Outcome data | 15* | *Cohort study*—Report numbers of outcome events or summary measures over time | N/A |
|  |  | *Case-control study—*Report numbers in each exposure category, or summary measures of exposure | N/A |
|  |  | *Cross-sectional study—*Report numbers of outcome events or summary measures | Cited on pages # 5-10.  Under the section ‘Results’.  Participants’ responses are the outcome measure. |
| Main results | 16 | (*a*) Give unadjusted estimates and, if applicable, confounder-adjusted estimates and their precision (eg, 95% confidence interval). Make clear which confounders were adjusted for and why they were included | Cited on pages # 5-10.  Under the section ‘Data Analysis’. |
|  |  | (*b*) Report category boundaries when continuous variables were categorized | N/A |
|  |  | (*c*) If relevant, consider translating estimates of relative risk into absolute risk for a meaningful time period | N/A |
| Other analyses | 17 | Report other analyses done—eg analyses of subgroups and interactions, and sensitivity analyses | None was carried out. |
| Discussion | | | |
| Key results | 18 | Summarise key results with reference to study objectives | Cited on pages # 10-14.  Under the section ‘Discussion’. |
| Limitations | 19 | Discuss limitations of the study, taking into account sources of potential bias or imprecision. Discuss both direction and magnitude of any potential bias | Cited on pages # 12-13.  Under the section ‘Discussion’. |
| Interpretation | 20 | Give a cautious overall interpretation of results considering objectives, limitations, multiplicity of analyses, results from similar studies, and other relevant evidence | Cited on pages # 10-14.  Under the section ‘Discussion’. |
| Generalisability | 21 | Discuss the generalisability (external validity) of the study results | Cited on pages # 13-14.  Under the section ‘Discussion’. |
| Other information | | | |
| Funding | 22 | Give the source of funding and the role of the funders for the present study and, if applicable, for the original study on which the present article is based | Cited on page # 14.  Under the section ‘Funding’. |

*Give information separately for cases and controls in case-control studies and, if applicable, for exposed and unexposed groups in the cohort and cross-sectional studies.

**References:**

1. Clarke V, Braun V. Teaching thematic analysis: Overcoming challenges and developing strategies for effective learning. The psychologist 2013;26(2)
2. O'cathain A, Murphy E, Nicholl J. The quality of mixed methods studies in health services research. Journal of Health Services Research & Policy 2008;13(2):92-98.
3. Kelley K, Clark B, Brown V, et al. Good practice in the conduct and reporting of survey research. International Journal for Quality in health care 2003;15(3):261-66.
