# Supplementary material for: Before the 2020 Pandemic: an observational study exploring public knowledge, attitudes, plans, and preferences towards death and end of life care in Wales
Source: BMC Palliat Care. 2021 Jul 20;20:116. doi: 10.1186/s12904-021-00806-2 (PMC8290392; doi:10.1186/s12904-021-00806-2)
Supplement: Supplementary file 2 — Survey Questionnaire. [file 12904_2021_806_MOESM2_ESM.doc]

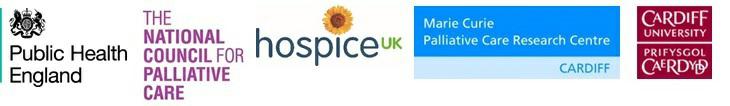


**SUPPLEMENTARY FILE 2:**

Survey on Public Attitude to Death and Dying in Wales

Part 1: What is this survey about?

We, a team of researchers from the Marie Curie Palliative Care Research Centre, want to find out about people’s attitude towards death and dying in Wales. We want to understand what people think and how they feel about death and dying. We want to understand what people know and if they have any preferences about issues related to death and dying. We would also like to know if you and your loved ones have made any plans around this.

Taking part in this survey is voluntary and you may choose not to answer some or any of the survey questions. There are no right or wrong answers.

We hope that the findings will help services to better understand the public perception around death and dying and develop informed service delivery. We also hope that this will encourage people to talk more about death and dying. We will publish the results of this study in the form of general reports and academic papers. Your name will not be used anywhere.

When using the internet, there can be a risk of compromising privacy, confidentiality and/or anonymity. We have taken the precaution of using the Bristol Online Survey Tool as it is known to be safe to use to minimise this risk.

We will use and manage all data in accordance with the General Data Protection Act 1998 currently used in the UK.

To request a Welsh language version of this survey or ask a question, please email islami@cardiff.ac.uk, Project Officer, or call 029 22510177.

We would like to thank you in advance for your help and time.

Part 2: Instructions for completion

- It should take about 10-15 minutes to complete.
- Please add any comments in the blank boxes that you feel are relevant or important.
- You may feel upset while filling out this questionnaire. You can complete it in more than one sitting if you like by clicking the 'finish later' link at the bottom of the page.
- You can browse the survey and change answers up to the point when you click the 'finish’ button.

Part 3: Consent to participate and how information will be used

**Consent:**

By agreeing to participate in this survey, you state that

- you live in Wales
- you have read and understood the information above and
- you are aged 18 or over.

You are also agreeing that we can include your responses and anonymised extracts of the questionnaire in future reports. Extracts may also be published in professional journals, presented at professional conferences and in educational settings. The information provided may be used for producing reports and academic papers and for future research.

**Do you agree to participate in this survey?**


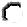
 Yes


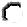
 No



Part 4: The survey

Section 1: You and your views

1. Please state your present circumstances from the drop-down menu.

| **Statement** |
| --- |
| I would consider myself to be in the last few years of my life. |
| I am a carer/ family member/ partner/ spouse/ friend of someone who is thought to be in the last few years of their life. |
| I am a bereaved carer/ family member/ partner/ spouse/ friend who lost a loved one in the last 5 years. |
| I am a professional working with people who are thought to be in the last few years of life. |
| I am a volunteer working with people who are thought to be in the last few years of life. |
| I am a health care professional. (clinical) |
| I am a health / social care professional. (non-clinical) |
| I am a health researcher/ an academic who has interest in the subject. |
| I am a member of public who has an interest in the subject. |

If other, please state ________________________________________

1. As a society, how much do we talk about death and dying in Wales?

- Too much
- About the right amount
- Not enough
- Don’t know

If not enough, how do you think this can be increased?

________________________________

1. To what extent do you agree or disagree with the following statements about End of Life Care?^1,2^

| **Statement** | **Strongly agree** | **Agree** | **Don’t know** | **Disagree** | **Strongly disagree** |
| --- | --- | --- | --- | --- | --- |
| There are adequate health and social care facilities available in Wales for people who are thought to be in the last 6 months of their life. |  |  |  |  |  |
| The religious/ spiritual needs of people who are thought to be in the last 6 months of their life are met adequately by the End of Life Care providers in Wales. |  |  |  |  |  |
| The cultural needs of people who are thought to be in the last 6 months of their life are met adequately by the End of Life Care providers in Wales. |  |  |  |  |  |
| People who are thought to be in the last 6 months of their life, can access End of Life Care facilities available in Wales. |  |  |  |  |  |
| People who are thought to be in the last 6 months of their life, can take part in the decision-making process of their health care in Wales. |  |  |  |  |  |
| In general, people who are dying in Wales are treated with dignity and respect by health and social care professionals. |  |  |  |  |  |

1. Do you think it is important to express your future health care preferences in advance?^3^

- Yes
- No
- Don’t know
- Other ________________________________________

1. What do you think is the most important benefit of expressing future health care preferences in advance of dying? Please tick one option:^3^

- Creating openness among family members/ friends/ care giver
- Lessening the burden to family members/ friends
- Reducing stress around planning and making decisions about my care
- Preventing me from having the treatment I don’t want
- Other _________________________________________

1. Do you believe in life after death?

- Yes
- No
- Don’t know
- Prefer not to say

Section 2: Your Feelings about death and dying

1. To what extent do you agree/ disagree with the following statements?

| **Statement** | **Strongly agree** | **Agree** | **Don’t know** | **Disagree** | **Strongly disagree** |
| --- | --- | --- | --- | --- | --- |
| I fear dying because I have to leave my loved ones. |  |  |  |  |  |
| I fear being helpless and dependent more than I fear death. |  |  |  |  |  |
| I fear the loss of my role in the family more than I fear death. |  |  |  |  |  |
| I fear the loss of my role in the community more than I fear death. |  |  |  |  |  |
| I am afraid to die for some other reason.  Please state _______________________________ |  |  |  |  |  |
| I am not afraid to die. |  |  |  |  |  |

1. How comfortable would you feel about discussing the following topics with your family and friends?^2^

| **Topics** | **Very comfortable** | **Comfortable** | **Don’t know** | **Uncomfortable** | **Very uncomfortable** |
| --- | --- | --- | --- | --- | --- |
| Death and dying in general |  |  |  |  |  |
| My End of Life wishes around treatment and care |  |  |  |  |  |
| Selecting someone who can decide for my treatment decision when I am unable to do it |  |  |  |  |  |
| My End of Life wishes around death (where I want to die, what will happen to my body/organ etc.) |  |  |  |  |  |
| My funeral arrangement |  |  |  |  |  |
| Arrangement of my financial affairs |  |  |  |  |  |
| Arrangement for my virtual possessions in the social media accounts (Facebook, Instagram etc.) |  |  |  |  |  |

1. How comfortable would you feel about the following situation?^2^

| **Situation** | **Very comfortable** | **Comfortable** | **Don’t know** | **Uncomfortable** | **Very uncomfortable** |
| --- | --- | --- | --- | --- | --- |
| Discussing my End of Life wishes with my doctors and nurses |  |  |  |  |  |
| A family member/ loved one discussing their End of Life wishes with me |  |  |  |  |  |

1. Is there anything that prevents you from talking about death and dying?

- Yes
- No

If yes, then please state

_________________________________________

Section 3: Your knowledge about death and dying

1. Please tick from the list below the terms that you are familiar with^4^

- Advance Directive
- Palliative Care
- Cardiopulmonary Resuscitation
- Artificial Hydration
- End of Life Care
- Do not Resuscitate Order (DNRO)
- Persistent Vegetative State
- Advance Care Plan
- Do Not Attempt Resuscitation (DNAR)
- Living Will
- Specialist Palliative Care
- Hospice Care
- Life Sustaining Treatment
- Do Not Attempt Cardiopulmonary Resuscitation (DNACPR).
- Power of Attorney

1. Please show to what extent you agree or disagree with the following statements about the availability of information or services regarding End of Life Care.^2^

| **Statement** | **Strongly agree** | **Agree** | **Don’t know** | **Disagree** | **Strongly disagree** |
| --- | --- | --- | --- | --- | --- |
| I know where to find information on how to plan in advance for my care at the end of life |  |  |  |  |  |
| If I’d like to make my End of Life Care plans, I know who among my friends or family I could discuss it with |  |  |  |  |  |
| If someone close to me were to die, I know where to find support |  |  |  |  |  |

Section 4: Your preferences about death and dying

1. Please show to what extent you agree or disagree with the following statements on your preferences about death and dying.^2,4^

| **Issue** | **Strongly agree** | **Agree** | **Don’t know** | **Disagree** | **Strongly disagree** | **Other** |
| --- | --- | --- | --- | --- | --- | --- |
| If I was severely ill with no hope of recovery, I would want to be kept alive at all costs. |  |  |  |  |  |  |
| If I was severely ill with no hope of recovery, my quality of life would be more important than the length of my life. |  |  |  |  |  |  |
| End of Life Care for older people should be as much of a priority for the NHS as care for people in any other stage of life. |  |  |  |  |  |  |
| End of Life Care for dying people should be as much of a priority for the NHS as care for people in any other stage of life. |  |  |  |  |  |  |
| My preferences should take priority over the wishes of my next of kin or my doctor’s advice. |  |  |  |  |  |  |

1. Please would you select the 3 most important services you think you might want to have during your final days of life?^1,2^

| **Services** | **Most important** |
| --- | --- |
| Having access to emergency care |  |
| Having access to other professionals for last minute concerns about my family or legal affairs |  |
| Having a trained carer nearby to help me and my family |  |
| Having privacy |  |
| Having my religious needs met. |  |
| Having my cultural needs met. |  |
| Having my spiritual needs met. |  |

1. Please would you select the 3 most important personal priorities you think you might want to apply during your final days of life?^2^

| **Conditions** | **Most important** |
| --- | --- |
| Being surrounded by my loved ones |  |
| Being surrounded by my personal things/pet |  |
| Being surrounded by other people around who are going through the same thing, to talk to and provide support |  |
| Being in my familiar surroundings |  |
| Being at my home |  |
| Being in a calm and peaceful atmosphere |  |
| Being symptom free |  |
| Being able to maintain my dignity and self-respect |  |
| Feeling safe |  |
| Being involved in decisions about my care |  |
| If I were not able to decide, being able to involve my family or person I trust to make decisions about my care. |  |

Section 5: Your plans

1. Please show how you agree or disagree with the following statements about the use of life-supporting technology (e.g. life-support machine or artificial ventilation etc.).

| **Statement** | **Strongly agree** | **Agree** | **Don’t know** | **Disagree** | **Strongly disagree** |
| --- | --- | --- | --- | --- | --- |
| I, as a patient, would be confident to get involved in the decision-making about using life-supporting technology. |  |  |  |  |  |
| Involving others in the decision-making of using life-supporting technology for my care would place extra burden on them. |  |  |  |  |  |
| Involving others in the decision-making of using life-supporting technology for my care would limit my privacy. |  |  |  |  |  |

1. Please select an option to express your position on the following statement:^2^

| **Statement** | **I have done this** | **I intend to do this** | **Don’t know** | **I do not intend to do this** | **I will not do this** |
| --- | --- | --- | --- | --- | --- |
| Formally expressing my future health care wishes and preferences (e.g. making a Living Will) |  |  |  |  |  |
| Talking to someone about my End of Life Care wishes |  |  |  |  |  |
| Talking to my doctors/ nurses about my End of Life Care wishes |  |  |  |  |  |
| Talking to someone about whether I want my body to be buried or cremated or donated |  |  |  |  |  |
| Making financial arrangement for the funeral |  |  |  |  |  |
| Making a decision on organ donation |  |  |  |  |  |
| Asking any family member/ friend whether they have made a living will |  |  |  |  |  |
| Asking any family member/ friend what type of care support they would want at the end of their lives |  |  |  |  |  |
| Asking any family member/ friend about their funeral wishes |  |  |  |  |  |
| Asking any family member/ friend where they would like to die |  |  |  |  |  |
| Asking any family member/ friend about their financial preparations for End of Life Care |  |  |  |  |  |

1. Please tell us anything you want to add about your feelings, wishes or experience in relation to care at the end of life.

____________________________________________________________________

Part 5: About you

**This section aims to collect information about yourself**

1. In which part of Wales do you live?

- North West
- North East
- Mid
- South West
- South East


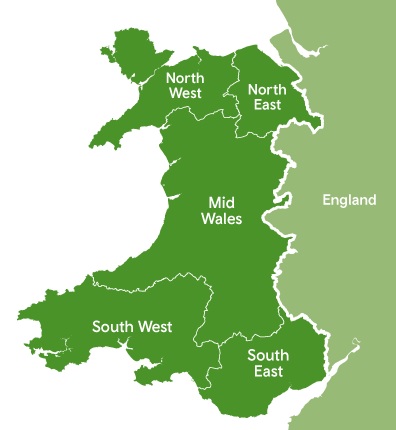


Please state only the first half of your postcode ____________________________________

1. Are you

- Single
- Married or with a partner
- Divorced
- Separated
- Widowed

1. Do you have any of the following conditions?

| Condition | Yes | No |
| --- | --- | --- |
| Chronic physical illness |  |  |
| Chronic mental illness |  |  |

1. Do you have any disability?

- Yes
- No

1. Do you have any religious belief?

- Yes
- No
- Prefer not to say

If yes, please select one of the following options

- Buddhism
- Christianity
- Hindu
- Islam
- Judaism
- Agnosticism
- Other ______________________________

1. If you have a religious belief, how important are your religious beliefs to the way you live your life?

- Highly important
- Important
- Unimportant
- Other ___________________

1. Which in the list below shows your highest level of education?

- Primary in the UK
- Primary outside the UK
- Secondary in the UK
- Secondary outside the UK
- Graduate or higher level in the UK
- Graduate or higher level outside the UK

1. Which in the list below shows the type of your accommodation?

- Private house/ flat
- Social/ council housing
- Sheltered housing
- Residential home
- Nursing home
- Long-stay hospital
- Hospice
- Other ___________________________

1. Which ethnic origin are you from?
2. White

- British
- English
- Irish
- Irish (Northern Ireland)
- Scottish
- Welsh
- Any other white

1. Mixed

- White and Black Caribbean
- White and Black African
- White and Asian
- Any other Mixed/ multiple ethnicity

1. Asian or Asian British

- Bangladeshi
- Indian
- Pakistani
- Chinese
- Any other Asian

1. Black or Black British

- Caribbean
- African
- Any other

1. Other ___________________
2. If you have moved from another country, how long have you been in Wales?

- Less than 5 years
- 5 to 10 years
- 10-15 years
- More than 15 years
- Not applicable

1. What is your gender?

- Male
- Female
- Other _______________________

1. What age group are you in?

- 18-24
- 25-34
- 35-44
- 45-54
- 55-64
- 65-69
- 70-74
- 75-79
- 80-84
- 85+

**Thank you for making a difference by completing this survey today.**

**References:**

1. Office for National Statistics. National Survey of Bereaved People ( VOICES ): England, 2015. [https://www.ons.gov.uk/peoplepopulationandcommunity/healthandsocialcare/healthcaresystem/bulletins/nationalsurveyofbereavedpeoplevoices/england2015not](https://eur03.safelinks.protection.outlook.com/?url=https%3A%2F%2Fwww.ons.gov.uk%2Fpeoplepopulationandcommunity%2Fhealthandsocialcare%2Fhealthcaresystem%2Fbulletins%2Fnationalsurveyofbereavedpeoplevoices%2Fengland2015not&data=04|01|IslamI@cardiff.ac.uk|10cfb325c76845c2d77308d89dc38e27|bdb74b3095684856bdbf06759778fcbc|1|0|637432810509351290|Unknown|TWFpbGZsb3d8eyJWIjoiMC4wLjAwMDAiLCJQIjoiV2luMzIiLCJBTiI6Ik1haWwiLCJXVCI6Mn0%3D|1000&sdata=dxHH2yIq6%2FsnvdTOjZ1rmiw2XOclkoVHi51%2FuZTRm9s%3D&reserved=0)
2. ComRes. Dying Matters Coalition – Public opinion on death and dying. 2016. <https://comresglobal.com/polls/dying-matters-coalition-public-opinion-on-death-and-dying/>
3. Best S, Tate T, Noble B, Smith R, Tuft J, Tracey N, et al. Research priority setting in palliative and end of life care: the James Lind Alliance approach consulting patients, carers and clinicians. BMJ supportive & palliative care. 2015;5(1):102-.
4. ComRes. Dying Matters Coalition – Public opinion on death and dying. 2015 <https://comresglobal.com/polls/national-council-for-palliative-care-public-opinion-on-death-and-dying/>
