# Supplementary material for: Before the 2020 Pandemic: an observational study exploring public knowledge, attitudes, plans, and preferences towards death and end of life care in Wales
Source: BMC Palliat Care. 2021 Jul 20;20:116. doi: 10.1186/s12904-021-00806-2 (PMC8290392; doi:10.1186/s12904-021-00806-2)
Supplement: Supplementary file 3 — Tables and Figures. [file 12904_2021_806_MOESM3_ESM.docx]

**SUPPLEMENTARY FILE 3:**

**Table S1: Socioeconomic and demographic characteristics of the study sample**

| Characteristics | n | % | % of Welsh population | Notes and references on % of Welsh population |
| --- | --- | --- | --- | --- |
| *Gender (n=2196)* |  |  |  | Year 2018^1^ |
| Female | 1674 | 76.2 | 49.0 |  |
| Male | 519 | 23.6 | 51.0 |  |
| Other | 3 | 0.1 | n/a |  |
| *Age group (n=2209)* |  |  |  | Year 2018^2^ |
| 18-24 | 70 | 3.2 | 8.4 | The ONS statistics report the group 20 to 24. |
| 25-34 | 183 | 8.3 | 16.2 |  |
| 35-44 | 303 | 13.7 | 14.5 |  |
| 45-54 | 441 | 20.00 | 17.6 |  |
| 55-64 | 642 | 29.1 | 16.5 |  |
| 65-74 | 472 | 21.3 | 14.8 |  |
| 75-84 | 94 | 4.2 | 8.6 |  |
| 85+ | 4 | 0.2 | 3.4 |  |
| *Ethnic origin (n=2201)* |  |  |  | Year 2018^3^ |
| White- Welsh | 944 | 42.9 |  |  |
| White- British | 905 | 41.1 |  |  |
| White- English | 198 | 9.0 |  |  |
| White- Irish | 14 | 0.6 |  |  |
| White- Irish (Northern Ireland) | 11 | 0.5 |  |  |
| White- Scottish | 20 | 0.9 |  |  |
| Any other white | 51 | 2.3 |  |  |
| All white ethic groups | 2143 | 97.4 | 95.0 |  |
| Asian or any other Asian | 14 | 0.6 |  |  |
| Black: African and Caribbean | 4 | 0.2 |  |  |
| Mixed | 23 | 1.0 |  |  |
| Other | 17 | 0.8 |  |  |
| Any other ethnic groups | 58 | 2.6 | 5.0 |  |
| *Moved from another country (n=1981)^4^* |  |  |  | Based on the 2011 census 5.5% of the Welsh population was non-UK. 22% of people living? in Wales come from England. |
| 10-15 years | 113 | 5.7 |  |  |
| 5 to 10 years | 98 | 4.9 |  |  |
| Less than 5 years | 100 | 5.0 |  |  |
| More than 15 years | 399 | 20.1 |  |  |
| Total moved from another country | 710 | 35.8 | 25.0 |  |
| Not applicable | 1271 | 64.2 | 75.0 |  |
|  |  |  |  |  |
| *Areas where Participants live (n=2201)^6^* |  |  |  |  |
| Mid | 300 | 13.6 | 6.5 |  |
| North East | 108 | 4.9 | 6.2 |  |
| North West | 151 | 6.9 | 16.1 |  |
| South East | 1062 | 48.2 | 48.8 |  |
| South West | 580 | 26.3 | 22.4 |  |
|  |  |  |  |  |
| *Highest level of education (n=2195)^7^* |  |  |  | England and Wales ONS 2011 |
| Graduate or higher level in the UK | 1522 | 69.3 | 18.8 |  |
| Graduate or higher level outside the UK | 41 | 1.9 |  |  |
| Primary in the UK | 43 | 2.0 | 23.0 | This reflects the percentage of people with no qualification. |
| Secondary in the UK | 568 | 25.9 | 28.0 | It combines level 1 and 2. |
| Secondary outside the UK | 21 | 1.0 |  |  |
|  |  |  |  |  |
| *Type of accommodation (n=2202)^8^* |  |  |  |  |
| Private house/ flat | 2086 | 94.7 | 69.5 |  |
| Residential home | 9 | 0.4 |  |  |
| Sheltered housing | 11 | 0.5 |  |  |
| Social/ council housing | 77 | 3.5 | 16.0 |  |
| Other | 19 | 0.9 |  |  |
|  |  |  |  |  |
| *Family status (n= 2199)* |  |  |  | 2,575,922 aged 16 and over in 2018 |
| Divorced | 206 | 9.4 |  |  |
| Married or with a partner | 1581 | 71.9 | 61.0 | It includes people who are divorced/separated but living with a partner now |
| Separated | 22 | 1.0 |  |  |
| Single | 254 | 11.6 | 23.9 | It includes people who are divorced/separated and live alone |
| Widowed | 136 | 6.2 | 15.0 |  |
|  |  |  |  |  |
| *Participants suffering from chronic physical illness (n=2166)^11^* |  |  |  | Statistics for England |
| Yes | 446 | 20.6 | 30.0 |  |
| No | 1720 | 79.4 |  |  |
|  |  |  |  |  |
| *Participants suffering from chronic mental illness (n=1992)^11^* |  |  |  | Statistics for England |
| Yes | 132 | 6.6 | 20.0 |  |
| No | 1860 | 93.4 |  |  |
|  |  |  |  |  |
| *Participants with any form ofdisability (n=2186)^12^* |  |  |  | 2015 Health survey |
| Yes | 324 | 14.8 | 33.0 |  |
| No | 1862 | 85.2 |  |  |
|  |  |  |  |  |
| *Religious belief (2196)^9^* |  |  |  | 2011 census (Ref 9) |
| Agnosticism | 52 | 2.4 |  |  |
| Buddhism | 35 | 1.6 | 0.3 |  |
| Christianity | 842 | 38.3 | 57.6 |  |
| Hindu | 4 | 0.2 | 0.3 |  |
| Islam | 6 | 0.3 | 1.5 |  |
| Judaism | 2 | 0.1 |  |  |
| Other | 118 | 5.4 | 0.4 |  |
| Prefer not to say | 113 | 5.1 | 7.6 |  |
| None | 1102 | 50.2 | 32.1 |  |
| 1. <https://statswales.gov.wales/Catalogue/Population-and-Migration/Population/Estimates/nationallevelpopulationestimates-by-year-age-ukcountry> 2. <https://www.ons.gov.uk/peoplepopulationandcommunity/populationandmigration/populationprojections/datasets/tablea25principalprojectionwalespopulationinagegroups> 3. <https://statswales.gov.wales/Catalogue/Equality-and-Diversity/Ethnicity/ethnicity-by-area-ethnicgroup> 4. <https://migrationobservatory.ox.ac.uk/resources/briefings/wales-census-profile/> 5. <https://www.ons.gov.uk/peoplepopulationandcommunity/populationandmigration/populationestimates/datasets/populationestimatesbymaritalstatusandlivingarrangementswales> 6. <https://webarchive.nationalarchives.gov.uk/20160108131743/http://www.ons.gov.uk/ons/dcp171778_290982.pdf> 7. <https://statswales.gov.wales/Catalogue/Population-and-Migration/Population/Estimates/Local-Authority/populationestimates-by-localauthority-year> 8. <https://statswales.gov.wales/Catalogue/Housing/Dwelling-Stock-Estimates/dwellingstockestimates-by-localauthority-tenure> 9. <https://gov.wales/sites/default/files/statistics-and-research/2018-12/151027-statistical-focus-religion-2011-census-executive-summary-en.pdf> 10. <https://www.kingsfund.org.uk/sites/default/files/field/field_publication_file/long-term-conditions-mental-health-cost-comorbidities-naylor-feb12.pdf> 11. <https://www.kingsfund.org.uk/sites/default/files/field/field_publication_file/long-term-conditions-mental-health-cost-comorbidities-naylor-feb12.pdf> 12. <https://gov.wales/sites/default/files/statistics-and-research/2019-02/160622-welsh-health-survey-2015-health-status-illnesses-other-conditions-en.pdf> | | | | |

**SUPPLEMENTARY FILE 3:**

**Table S2: Relevant quotations on barriers and enablers to talking about death and preferences around EoLC**

| **Topic** | **Theme** | **Quotations examples (Participant ID number)** |
| --- | --- | --- |
| Barriers to talking | Personal emotions and values | “Just generally scared and uncomfortable especially as my Mum has dementia and is in last years of life as it makes me realise I am closer to my own demise now” (PID 1999 )  “Talking about death with others can be hard if they find it a difficult subject. I've experienced it quite a bit in the past few years so for me it feels like a normal subject. For others it can be upsetting and so often I try not to talk about it for fear of upsetting them” (PID 048)  “If there are young children present, i feel uncomfortable to talk about death” (PID 825) |
|  | Social perception and practice | “People just don't want to!” (PID 036)  “its perceived to be bad taste or impolite. how do we raise such a topic in day to day life. its seen as morbid and not something people want to think of. people aren’t comfortable talking about their own or loved ones mortality ” (PID 839) |
|  | Lack of opportunity and support | “I don't have any family and only really good friend who would carry out my wishes but is the same age as me so may die before I do” (PID 160)  “The ability of health and care professionals to discuss death and dying” (PID 536) |
| Enablers to talking | Normalising talk and creating openness | “We need to start the conversation and not shy away from using the proper words rather than euphemisms” (PID 139)  “Getting more stories heard and opening debates and conversations about the subject”(PID 137)  “Most people's perception of dying has a lot of inaccuracies included and is often based on years of religious teaching. Unless we have experience of death in the family, we do not really know much about it. Not everyone may wish to go against what they were told as a child but correct information needs to available if needed especially as we now have the internet”(PID 245)  “People are scared of dying and it is seen as morbid even though it’s a part of being alive!! As a family we talk about dying as it’s inevitable. Death is the hardest for the ones left behind. Counselling helps but the person needs to be ready to talk about painful feelings and society sees having feelings as a weakness which is wrong. Society needs to know it’s okay to feel sad and angry it’s a natural process of grief it’s not a weakness”(PID 656) |
|  | Means and actions for normalising talk | “More news items regarding things such as donor cards, different options for burial / cremation but with a less serious and more plain speaking tone. Showing choices that people have made without the tears”(PID 010)  “Talking to everyone, via social media or other campaign about having a 'good' death, so it's in people thoughts”(PID 091)  “What if... Just like opting in or out to organ donation, you do the same with your remains; donate to science, burn, bury or other (to be mentioned in last will or some-such)? ”(PID 482)  “Education both schooling and health education ... medical and clinical staff don’t get enough exposure to ‘How to talk about dying’ through training”(PID 021)  “More education or learning at a younger age so the subject is more 'normal' rather than taboo”(PID 052)  “Death Cafe or similar, bring people together to explore wide range of topics around death. Public campaign to encourage everyone to share end of life wishes with family and friends Easy way to make an advance directive to be held by GP and NHS hospitals, joint IT would simplify”(PID 510) |
| Preferences for EoLC: Services and personal priorities | Biological support | “Having pain relief freely available”(PID 1838)  “Having a Registered Nurse available with a specialist qualification in EOL care”(PID 022)  “Having the right to say when I want to die if I have a poor prognosis - Euthanasia is the act of deliberately ending a person's life to relieve suffering”(PID 259) |
|  | Psycho-social support | “More private hospital rooms. Dying on a ward is unacceptable”(PID 934)  “Being treated with respect after I die”(PID 973)  “Assurance that my stated wishes will be observed”(PID 1089)  “Making sure that my family has easy access to counselling not just family counselling but individual as we all affect people individually”(PID 1296)  “Having emotional needs met. Having access to mental health professional support”(PID 1197)  “being safe”(PID 1365) |
|  | Place and environment | “Being in a peaceful place”(PID 1964)  “Being around loved ones”(PID 326)  “I would want access to my beloved dog”(PID 154)  “Being close enough to friends for them to visit”(PID 419)  “Being able to say goodbye to those I love”(PID 895)  “To be at home with good calm care”(PID 159) |
| Fear of Death |  | It is the process of dying, being in pain or out of control not the act of being dead that is scary to me (PID 339)  I don’t fear death but the manner of dying (PID 32)  I fear powerlessness I fear pain I fear losing my mind (PID 27)  If it happened now - where would my children live? (PID 404)  I'm still afraid to die, it's the unknown that causes the fear I believe (PID 48) |

**SUPPLEMENTARY FILE 3:**

**Fig S1: The extent to which participants agreed/disagreed with statements about fear of death and dying**

**SUPPLEMENTARY FILE 3:**

**Fig S2: Participants’ preferences about death and dying and EoLC**

**SUPPLEMENTARY FILE 3:**

**Fig S3: Participants’ attitude towards discussing death and dying related topics**

**SUPPLEMENTARY FILE 3:**

**Fig S4: Participants’ attitude towards life-supporting technology**

**SUPPLEMENTARY FILE 3:**

**Fig S5: Participants’ knowledge around the availability of information or services around EoLC**
